# Supplementary material for: Effects of curvature on growing films of microorganisms
Source: Biophys J. 2025 Apr 7;124(10):1609–17. doi: 10.1016/j.bpj.2025.04.003 (PMC12242409; doi:10.1016/j.bpj.2025.04.003)
Supplement: Document S1. Figures S1–S3 and supporting material [file mmc1.pdf]

Biophysical Journal, Volume *124*

Supplemental information

Effects of curvature on growing films of microorganisms

Yuta Kuroda, Takeshi Kawasaki, and Andreas M. Menzel

Supporting Material for "Effects of curvature on growing films of microorganisms"

Yuta Kuroda
Institut für Physik, Otto-von-Guericke-Universität Magdeburg, Universitätsplatz 2, 39106 Magdeburg, Germany and Department of Physics, Nagoya University, Nagoya 464-8602, Japan

Takeshi Kawasaki
Department of Physics, Nagoya University, Nagoya 464-8602, Japan

Andreas M. Menzel
Institut für Physik, Otto-von-Guericke-Universität Magdeburg, Universitätsplatz 2, 39106 Magdeburg, Germany

1. DEPENDENCE OF THE RADIUS OF THE COLONY ON THE CELLULAR CURVATURE AND THE THRESHOLD DIVISION LENGTH

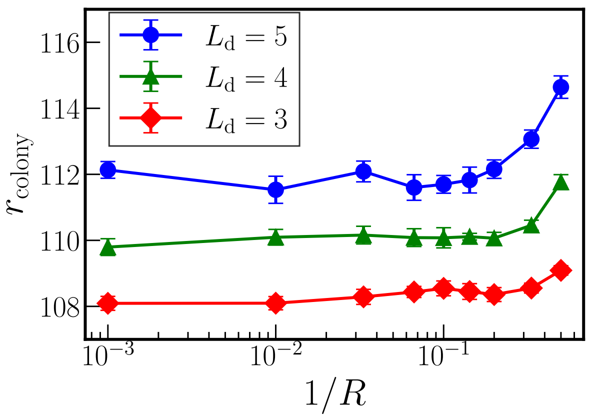

FIG. S1. Radius of the colony r_colony as a function of the curvature of the individual cells $1/R$ for different threshold division lengths L_d.

Figure S1 depicts the radius of the entire colony r_colony for our final configurations at which we stop our simulations as a function of the curvature of the individual cells $1/R$. Results are displayed for the three threshold division lengths $L_\mathrm{d} = 5$, 4, and 3. Ensemble averages are taken over 10 different samples for each data point. For all values of L_d, the radius r_colony increases as the curvature increases for elevated values of $1/R$. This is natural as the colony of curved cells contains more gaps compared to the situation of straight constituents.

2. SPATIO-ORIENTATIONAL COUPLING FOR THRESHOLD DIVISION LENGTHS $L_\mathrm{d} = 3$ AND 4

In the main text, we show the histograms for $\theta_i - \gamma_i$ as a measure of spatio-orientational coupling for the threshold division length $L_\mathrm{d} = 5$. This is the largest threshold division length considered in our simulations, see Fig. 4 in the main text. θ_i measures the individual angular orientation of each cell i, while γ_i quantifies its angular position within the colony, see Fig. 4(a) in the main text. Here, we include in Fig. S2 the corresponding histograms of $\theta_i - \gamma_i$ for the smaller division lengths $L_\mathrm{d} = 3$ and 4. Obviously, even for these, we find inhomogeneous unimodal distributions for the cellular curvatures $1/R = 0.10$ and 0.33. Thus, again spatio-orientational coupling is observed. However, the distributions are flatter for the smaller threshold division lengths $L_\mathrm{d} = 3$ and 4.

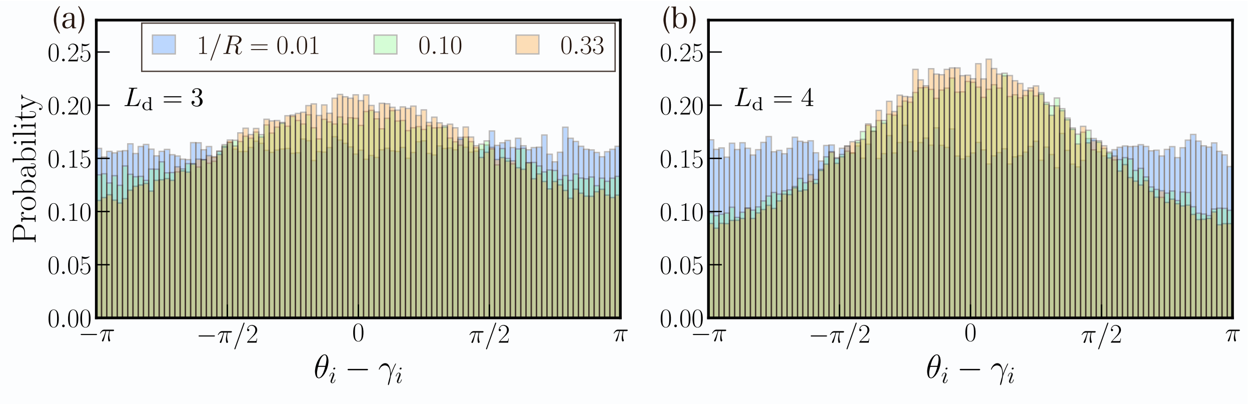

FIG. S2. Histograms of $\theta_i - \gamma_i$ as a measure for spatio-orientational coupling within the colony for threshold division lengths (a) $L_d = 3$ and (b) $L_d = 4$.

3. EFFECTS OF THE THRESHOLD VALUE ON THE AVERAGE DOMAIN AREA $\langle A \rangle$

In the main text, we classify two objects i and j as belonging to the same domain, if they feature a contact force and if they roughly show identical orientation. For the latter measure, we define a threshold (upper bound) for the deviations in their orientation angles θ_i and θ_j. Specifically, we require $|\theta_i - \theta_j| < 0.10$. Objects satisfying both requirements belong to the same domain, and we calculate the domain area $\langle A \rangle$ averaged over the colony.

We here demonstrate that our results are qualitatively robust with respect to the precise choice of this threshold value. Accordingly, Fig. S3 shows the average domain area $\langle A \rangle$ as a function of curvature $1/R$ calculated for three different threshold values 0.07, 0.10, and 0.13. Quantitative changes concerning the height of these curves result. However, their qualitative behavior is not altered. We hence conclude that the choice of the precise value associated with this threshold does not qualitatively affect the results displayed in the main text.

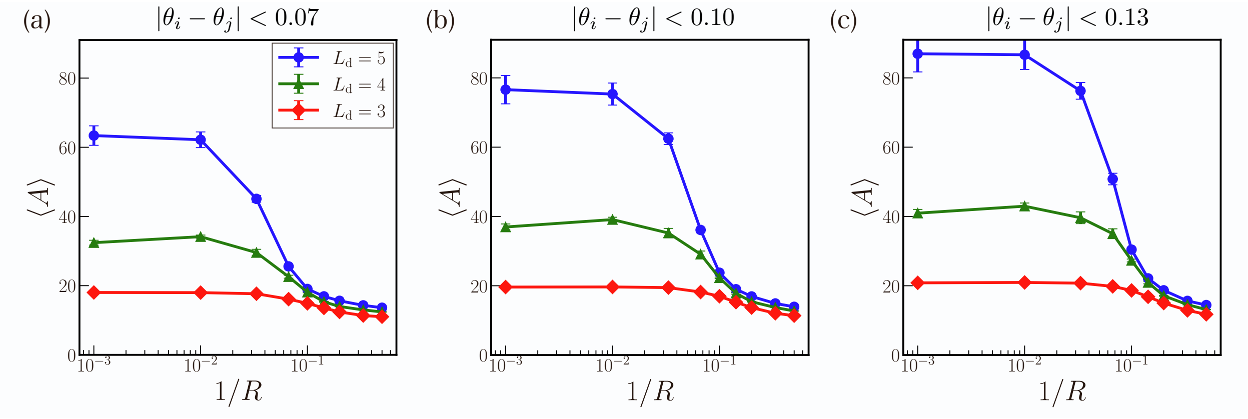

FIG. S3. Average domain area $\langle A \rangle$ for different threshold values (a) 0.07, (b) 0.10, and (c) 0.13 for the deviation $|\theta_i - \theta_j|$ between the orientations θ_i and θ_j of two objects i and j. These threshold values determine whether two nearby objects can be assigned to the same domain. Panel (b) is the situation included as Fig. 3(a) in the main text.
